# P-1022. Modeling the Impact of Sampling Intensity on Observing C. difficile Transmission in Healthcare Settings

**DOI:** 10.1093/ofid/ofaf695.1218

**Published:** 2026-01-11

**Authors:** Savannah Curtis, Sankalp Arya, Cristina Lanzas

**Affiliations:** North Carolina State University; North Carolina State University; North Carolina State

## Abstract

**Background:**

Whole genome sequencing (WGS) is increasingly used to investigate healthcare-associated infections, yet prior studies have only been able to link a small percentage of *C. difficile* cases. It remains unclear whether this is due to low sampling intensity or because transmission events are occurring outside of monitored healthcare settings.

**Methods:**

We developed a stochastic transmission model integrated with an observation model to simulate *C. difficile* spread and case detection under varying sampling intensities. The model was originally fitted to data from a six-month prospective cohort study with weekly active surveillance sampling. Retrospective analyses of the data were combined with prospective simulation-based analyses using synthetic testing datasets at three sampling intensities (standard, intermediate, and intensive). Transmission trees were constructed to examine transmission events’ temporal, spatial, and network-scale dynamics.

**Results:**

At the standard sampling level (weekly testing), an estimated 21.9% of cases and only 6.8% of transmission pairs were observed. Increasing sampling frequency to daily testing improved observability to 65.0% of cases and 55.2% of transmission pairs. Transmission events occurred across diverse time scales, with 95.5% of modeled transmission trees containing at least one pair separated by four or more weeks, highlighting the pathogen's persistence in healthcare environments. Cluster analyses revealed that over half of transmission trees contained at least one cluster linking 15 or more new colonizations.

**Conclusion:**

Our findings demonstrate that substantial transmission occurs but that weekly sampling is insufficient to capture most *C. difficile* transmission events. Enhanced sampling intensity substantially improves the observability of cases and transmission pairs, underscoring the need for more comprehensive surveillance protocols. Additionally, modeling approaches can augment genomic epidemiology by illuminating unobserved transmission pathways, allowing for improved tree reconstruction.

**Disclosures:**

All Authors: No reported disclosures

